# Ecological Roles of Lichens as Monitors of a Changing Global Environment

**DOI:** 10.3390/biology15060478

**Published:** 2026-03-17

**Authors:** Melanie Bih Beng Fung, Alexander G. Paukov, Ji-Wei Yuan, Hai-Xia Wang, Bo-Ya Cui, Hua-Jing Liu, Qiang Ren

**Affiliations:** 1 Zhejiang Key Laboratory for Restoration of Damaged Coastal Ecosystems, School of Life Sciences, Taizhou University, Taizhou 318000, China; bengmelanie1@gmail.com (M.B.B.F.);; 2 Institute of Natural Sciences and Mathematics, Ural Federal University, Lenina av., 51, Ekaterinburg 620000, Russia; 3 Ecological and Environmental Monitoring Center of Zhejiang Province Taizhou City, Taizhou 318000, China

**Keywords:** airborne contaminants, atmospheric deposition, biomonitoring, climate change, lichens, symbiosis

## Abstract

Lichens represent a fundamental symbiotic association between fungi and photosynthetic organisms, such as algae or cyanobacteria, and are widely regarded as sensitive indicators of environmental change. Lichens’ capacity to colonize a wide range of ecological niches is attributed to their distinctive physiological characteristics, notably, their lack of protective cuticles and ability to uptake water and nutrients directly from the atmosphere. Concurrently, lichens are highly vulnerable to airborne contaminants, making them critical bioindicators of air quality. However, the survival of lichens is increasingly influenced by intensifying global change via agriculture, industrial activities, and vehicular emissions. Organic and inorganic pollutants can adversely affect lichen physiology by inducing pigment degradation, disrupting membranes, and altering lichen diversity. The synergistic stressors associated with global change, such as increasing temperatures and shifts in precipitation regimes, exacerbate the effects of atmospheric deposition and oxidative stress on lichens. Here, we present existing knowledge on lichens’ ecological functions, elucidate the mechanisms underlying their sensitivity to air pollution, and assess their utility for environmental monitoring amid accelerating global change. By recognizing lichens as dynamic ecological indicators, we underscore their dual role in sustaining ecosystem processes amidst rapid global change.

## Simple Summary

Lichens are complex, self-contained ecosystems formed by the symbiotic relationship between fungi and photosynthetic organisms, such as algae or cyanobacteria, which can signal when the environment is changing or becoming polluted. Because they lack a protective skin layer, they can absorb water directly from the air or soil. While this helps them live almost everywhere, it also makes them extremely sensitive to pollution. For this reason, we review how lichens act as biological indicators, or “bioindicators,” of environmental quality. Today, pollution from farming, factories, and cars is threatening their survival. These pollutants damage lichens by destroying their pigments (turning them white) and breaking down their cellular structures. The problem is worsening due to rising temperatures, changing rainfall patterns, and pollution. Our study found that lichens act as an early warning system: when they start to disappear or their abundance and diversity decline, it signals that the environment is becoming too toxic for their survival, growth, and activities.

## 1. Introduction

**Figure 1:**
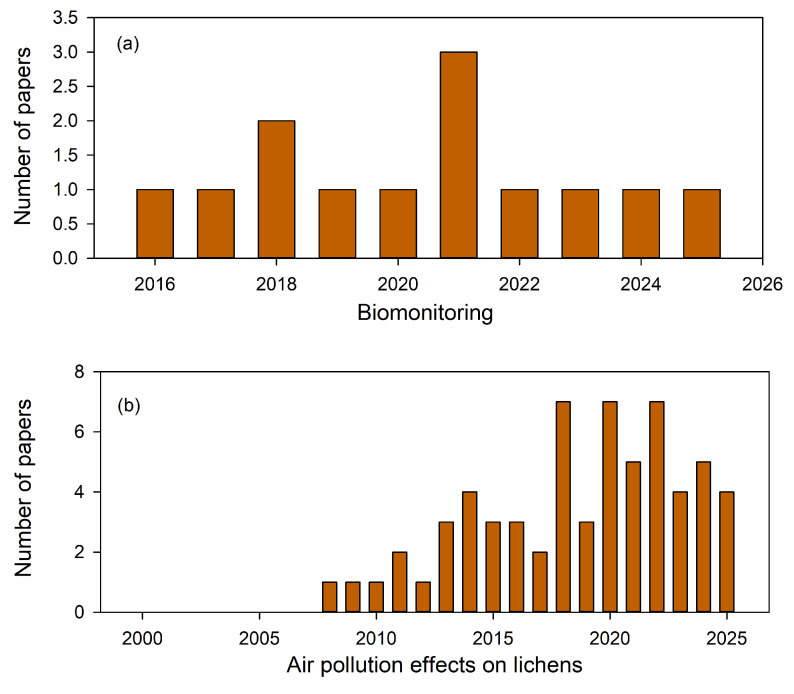

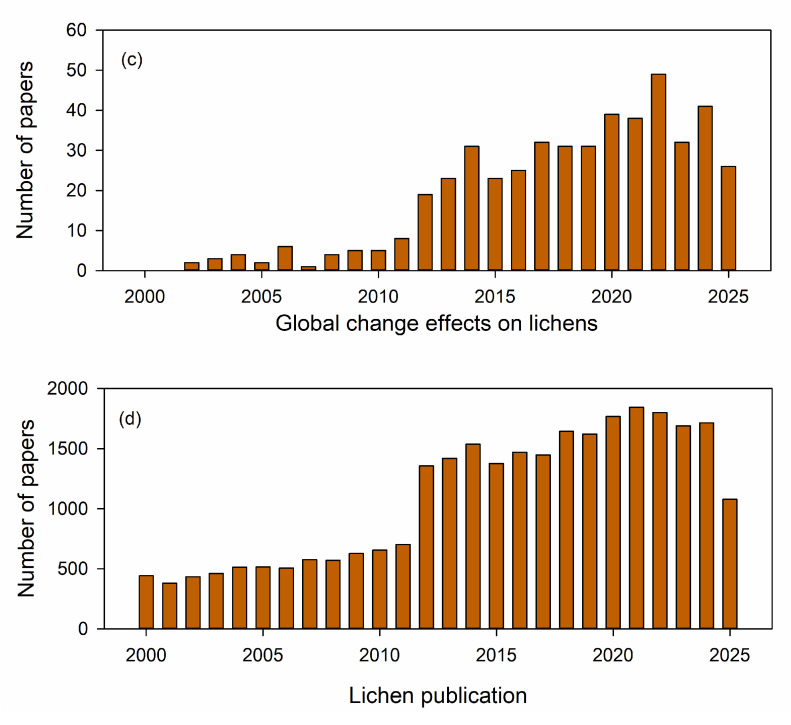
Number of papers published on (**a**) lichen biomonitoring, (**b**) air pollution effects on lichens, (**c**) global change effects on lichens, and (**d**) overall lichen publications per year. All graphs were produced from 14 September 2025 by searches of the ISI Web of Science database for a combination of key terms from date range of 1 January 2000–31 December 2025. Search began with: lichen, and then filtered for global change, air pollution, and then biomonitoring. Only original research articles were considered, excluding all forms of review articles.

Lichens account for approximately 20% of known fungi species [1,2], forming symbiotic associations with an alga or cyanobacterium (photobiont) in most terrestrial ecosystems [1,3,4,5]. The unique adaptability and defense mechanisms exhibited by lichens enable them to colonize and persist across extreme environmental gradients [6,7], including arid deserts, montane habitats, and nutrient-poor soils [5,8]. Lichens play pivotal ecological roles, substantially affecting soil biogeochemical properties and the atmospheric environment [9]. The global environment is undergoing significant transformations due to anthropogenic disturbances, including industrial activities, agricultural practices, and the expansion of urban communities. Consequently, there has been a heightened focus on the ecological sensitivities of lichens as environmental biomonitoring agents. The unique characteristics of lichens, including their diversity and intricate biological structures, coupled with their slow growth rates [3,9] and the capacity to absorb atmospheric substances directly in the absence of a protective cuticle, render them exceptional ecological indicators for monitoring environmental quality [10,11,12], especially in the context of a changing global environment (Figure 1).

Lichens serve as primary components of many vital biogeochemical processes, supporting and sustaining soil health and productivity [9,13]. For example, lichens play essential roles in the ecological restoration of barren surfaces by stabilizing soils and forming microhabitats. These microhabitats facilitate the establishment and growth of many organisms, including plant species. In forest ecosystems, lichens promote nutrient cycling by fixing atmospheric nitrogen into soils [14,15]. These lichen-mediated fixed nutrients are particularly essential for plants in boreal and temperate ecosystems where nitrogen availability can be limiting, thereby affecting plant growth [16,17,18]. Additionally, their sensitivity to microclimatic variables is crucial for sustaining local biodiversity and ecological equilibrium [19]. By influencing hydrology, shading, and surface albedo, lichens significantly modulate their environments and support environmental resilience [20,21].

Human activities, including agriculture and industrialization, are among the critical and widespread environmental challenges of the 21st century [22,23], threatening ecosystems worldwide. Human-driven inputs, such as reactive nitrogen dioxide, ozone, heavy metals, and airborne microplastics, have profound consequences on biochemical cycles and ecosystem health [24,25,26,27]. In particular, the increasing accumulation of these substances in the atmosphere can lead to acidic rain, which significantly affects microbial communities [28,29]. These contaminants also disrupt ecosystem functions such as nutrient cycling, decomposition, and primary productivity [29]. As such, eutrophication or high nutrient availability within oligotrophic ecosystems [30,31] has altered both the diversity and species composition of lichens and their associated communities or mycobiomes (e.g., bacteria) across numerous habitats [32,33,34,35]. Such ecological consequences of chronic environmental pollution have become increasingly evident, requiring urgent remedies through experimental exploration to enhance the sustainability and management of global ecosystems. As a result, previous studies indicate that lichens can serve as bioindicators of environmental quality [10,11,36]; however, more studies are still needed to advance understanding of this prospect. Lichen physiology and ecological traits underpin their potential as important indicators of environmental quality. Thus, the lack of roots and protective barriers [3,5] enables lichens to absorb water and nutrients directly from the atmosphere, making them highly sensitive and vulnerable to airborne and other contaminants [5]. Consequently, changes in lichen diversity, abundance, and morphology have been extensively used to infer pollution levels and types [36,37]. Specifically, epiphytic lichens have been widely used for monitoring air quality [38]. The responsiveness of lichens to airborne pollutants, such as sulfur dioxide, nitrogen compounds, and heavy metals, provides a cost-effective, integrative tool for environmental assessment. As concerns about environmental pollution escalate amid ongoing global environmental change, lichens can serve as vital biocontrol agents for environmental management. Therefore, this review discusses the role of lichens as natural bioindicators, with particular emphasis on their physiological adaptations to human-driven inputs, e.g., heavy metals, microplastics, and nutrients, the underlying mechanisms, and their sensitivity. Furthermore, we examine lichens within the framework of global environmental change and explore methodological advancements in lichen biomonitoring.

## 2. Lichens as Natural Bioindicators: Mechanisms and Sensitivity

### 2.1. Physiological Adaptations of Lichens to Heavy Metals

**Figure 2:**
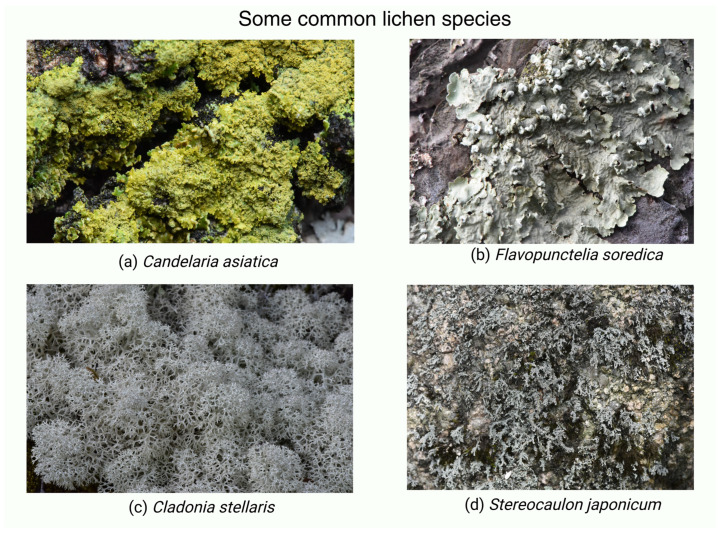
Some common lichen species.

Lichens are distinguished by their exceptional capacity to colonize or inhabit extreme environments [39,40], including heavy metal (e.g., lead, cadmium, and zinc) contaminated soils [41,42]. This ability is attributed to their remarkable tolerance to heavy-metal stress, facilitated by both physiological and biochemical adaptations [43,44]. For instance, recent studies have demonstrated that exposure of lichens to heavy metals, including arsenic, significantly compromises their chlorophyll and protein content [41,44,45,46,47,48,49]. Importantly, lichens absorb heavy metals predominantly through atmospheric deposition because they lack root systems and rely on surface absorption mechanisms [42,43]. Their high surface-area-to-volume ratio underlies their capacity to absorb and retain metal contaminants from rain, dust, and air. The fungal component of the lichen’s cell walls facilitates metal binding to the thallus owing to the presence of negatively charged functional groups, which attract and immobilize metal cations (Figure 2, Table 1).

Indeed, these adaptation mechanisms enable lichens to survive, accumulate, and detoxify metals, underscoring their usefulness as bioindicators of environmental pollution in many ecosystems, including remote environments such as Antarctica [50,51,52,53,54]. Among the primary mechanisms by which lichens mitigate metal toxicity are extracellular and intracellular detoxification processes [55,56,57]. During extracellular detoxification, metal contaminants are sequestered on the fungal cell wall as insoluble compounds, such as oxalates and phosphates, thereby reducing their bioavailability [55]. Intracellularly, lichens employ chelation to sequester metals, preventing interference with vital metabolic functions [58]. Despite their robust adaptive mechanisms and defense strategies against heavy-metal-induced oxidative stress, reactive oxygen species generated by heavy metals can inflict substantial damage to cellular components, including lipids and proteins [29,44].

Nevertheless, lichens possess intrinsic capabilities to regulate and neutralize oxidative stress through an antioxidant defense system composed of enzymatic components such as catalase and peroxidase [59]. Additionally, non-enzymatic antioxidants, such as carotenoids and ascorbic acid, play crucial roles in mitigating reactive-metal-induced stress in lichens [59,60]. It has been documented that certain lichen species augment the production of these antioxidants in response to metal stress, thereby enhancing their survival in metal-laden environments [60,61]. Moreover, lichen metabolites have also been shown to mitigate the effects of metals by forming chelates that inactivate these compounds [20,42,62].

**Table 1:** Evidence of environmental contaminant effects on lichen communities.

Lichen Species	Location/ Ecosystem	Contaminants	Exposure Levels or Quantities	Observed Effects or Results	Reference
*Stereocaulon vesuvianum* and *Parmelia saxatilis*	Montane area	Nitrogen (N) deposition	3–40 Nakg ha^−1^ year^−1^	Cephalodium abundance in *S. vesuvianum*, and ^15^N concentration in *S. vesuvianum* and *P. saxatilis*, were strongly negatively correlated with N deposition and particularly with dry deposited N	[15]
*Evernia prunastri*	Urban area	Airborne microplastics	94.6% were classified as fibers and 5.4% as fragments	Microplastic deposition in Milan to be in the range of 43–119 MPs m^2^/d	[26]
*Cladonia* and *Xanthoria*	airborne	Microplastics and Mesoplastics	97% fibers, and 3% fragments	Gradient in the number of microplastic fibers across the sites, with increasing accumulation of microplastics from the natural site (*n* = 58) to the urban site (*n* = 116)	[27]
*Candelaria concolor*, *Candelariella vitellina*, *Collema furfuraceum*, *Melanelixia glabra*, *Phaeophyscia orbicularis*	Urban center	Atmospheric nitrogen (N)	Variable	Eutroph abundance correlated negatively with trunk bark pH, exactly the opposite of virtually all previous studies of eutroph behavior	[33]
*Cetraria islandica*	Mediterranean	Al, Cr, Li, and Mg	>72% for Cd, >58% for Hg and >55% for Pb	Al, Cr, Li, Mg, significantly decreased with elevation, while the concentration of Cu and Fe is significantly higher on the eastern slope	[37]
*Candelariella aurella*, *Lecanora muralis*, and *Lecidea fuscoatra*	Mining site	Zn, Pb, Cd, and Ni	-	A host substrate colonized by studied lichens shows a broad spectrum of heavy metal contents; the ranges were as follows: Zn = 0.02–9.27%, Pb = 0.02–5.82%, Cd = 0.61–1625 lg g^−1^, and Ni = 5.65–883 lg g^−1^	[41]
*Hypogymnia physodes* and *Cladonia furcata*	Forest	As	Exposed to 0, 0.1, 1 and 10 lg mL^−1^ arsenate	In *H. physodes*, which contained higher amounts of arsenic compared to *Cl. furcata*, total glutathione content significantly decreased in samples exposed to 10 lg mL^−1^ As(V), whereas in *Cl. furcata* a significant increase was observed	[43]
*Candelaria*, *Cetrelia*, *Dirinaria*, *Heterodermia*, *Myelochroa*, *Parmotrema*, *Phaeophyscia*, and *Punctelia*	Jeju Island	As and Cu	Variable	Arsenic exerted a significant impact on chlorophyll degradation and protein content	[44]
*Xanthoparmelia camtschadalis*	Road side	Heavy metals	Variable	Metal concentrations peaked after 9-month exposure at exposure sites near roads, suggesting that the emissions near the roads accelerated the accumulation of metals	[45]
*Physcia aipolia*, *P*. *tribacia*, *Xanthoria elegans*, *X*. *mandschurica*, *Xanthoparmelia camtschadalis*, *X*. *tinctina*	Atmospheric elemental deposition	Heavy metals	variable	The elemental concentrations in lichens were both species- and element-specific, highlighting the importance of species selection for biomonitoring air pollution using lichens	[46]
*Phaeophyscia hirtuosa*, *Candelaria fibrosa*	Anthropogenic emissions	Heavy metals	variable	The results demonstrate that lichen elemental compositions are highly influenced by both their natural environment and anthropogenic input	[47]
*Xanthoria parietina*, *Physconia grisea*, and *Physcia adscendens*	Laboratory	Cd, Pb, and Zn	Variable	The Cd-exposed lichens grown in Italy produced significantly larger amounts of phytochelatins than the same species grown in Poland	[58]
*Ramalina* and *Xanthoria*	Mercury storage center	Hg	60 and 1000 ng m^−3^ and 153 and 1080 ng g^−1^	The mercury concentration in the gas phase in Flix was higher than that found in Las Cuevas	[63]
*Ramalina duriaei*	HaZorea Forest	Pb, Cd, Ni, Fe, S, Mg, Na, Ca, and K	Variable	Damaged to the cell membrane	[64]
*Physcia adscendens*	Urban area	Particulate matter	PM_10_ and PM_2.5_	Lichens from highly polluted sites also had higher photosynthetic efficiency, indicating the strong adaptability of this species to air pollution	[65]
*Hypogymnia physodes*	Forest	Cu and Mn	Variable	Increased Cu^2+^ and Mn^2+^ tolerance stimulated the evolution of lichen substances in *H. physodes*	[66]
Epiphytic lichens	Seven European cities	Air contaminants	Variable	Taxonomic metrics were better explained by air pollution, as expected, while climate did not surpass air pollution in any of the trait-based metric groups	[67]
Crustose and foliose lichens	Shiraz City, southwest Iran	Microplastics (MPs) and micro-rubbers (MRs)	MPs and MRs abundance overall was <1 g^−1^ and <0.1 g^−1^	Among the lichens, members of the genus *Acarospora*, with their areolated form, appeared to act as the most suitable biomonitors for airborne microplastics or microrubbers	[68]
*Cladonia* spp.	Contaminated slag dumps	Zn, Pb, Cd, and As	-	Each dump can be characterized by highly unfavorable habitat conditions and extreme heavy metal contamination	[69]
Cyanolichens, pendant and shrubby forage lichens	Forest	Nitrogen (N) and Sulfur (S)	Low, moderate, and high ecological risk	Cyanolichens and forage lichens abundance dropped rapidly with N and S deposition, declining 80% by 6.6 kg N and 11 kg S and 10 kg N and 13 kg S ha^−1^ y^−1^, respectively	[70]
*Chrysothrix candelaris*, *Graphis scripta*, *Pertusaria leucosorodes*, *Dirinaria consimilis*, *D. aegialita*	Urban center	Cd, Cr, Cu, Fe, Ni, Pb, and Zn	Variable	It was observed that the lichen diversity was less near heavy traffic locations and industrial areas	[71]
*Usnea longissima*, *U. luridorufa*, *U. dasopoga*, and *U. betulina*, *Ramalina calicaris* var. japonica	Shennongjia Nature Reserve (China) and Laboratory	Nitrogen (N)	10 to 20, and reached a maximum at 50 kg N ha^−1^ a^−1^	The N addition also led to the N:P ratios of five lichens increased from about 10 to 20, and reached a maximum at 50 kg N ha^−1^ a^−1^	[72]
*Hypogymnia physodes*	Nature Reserve	Al, As, Cd, Co, Cu, Ge, Fe, Mn, Mo, Ni, Pb, Sn, Ti, V, and Zn	Within permissible limits	An excess of moisture in ecotopes located near rivers and swamps increased the gross concentration of separate elements in the lichens	[73]
*Hypogymnia physodes*, *Parmelia sulcata*, *Pseudevernia furfuracea* *Usnea hirta*	Atmospheric pollution	Metal pollution (29 elements)	Variable	Metal accumulation varies with element and the species investigated	[74]
*Xanthoria parietina*	Mining site	Heavy metals	Variable	The concentration pollution maps and G-score maps of Pb and Ba were similar, and this was an indication of the vehicle emissions	[75]
*Ramalina fastigiata*	Industrial area	Polycyclic aromatic hydrocarbons (PAHs)	247 and 841 ng/g (dry weight)	A significant positive linear correlation was found between the concentrations of low molecular weight PAHs in lichens and the amounts accumulated in passive air samples	[76]
*Evernia prunastri*	Landfill site	trace elements	Variable	Increasing nitrogen availability decreased non-nitrophilous lichen species, corresponding to higher levels of eutrophication	[77]
*Flavopunctelia soredica* and *Rhizoplaca chrysoleuca*	Roadside emission	Heavy metals	Variable	The two lichen species exhibited roughly similar spatio-temporal patterns in element concentrations, with significant increases observed in relation to the distance to the road and exposure time	[78]
*Usnea antarctica*	James Ross Island, Antarctica	Toxic elements	Variable	*U. antarctica* preferentially accumulates Fe and atmospheric deposition in the cortical layer, while Cr, Cu, Ni, Zn, and Pb exhibited a gradient from the cortex to the medulla	[50]
*Usnea aurantiaco-atra* and *U. antarctica*	Shetland Island, Antarctica	Cd, Zn, Pb, Cu, Mn, and Fe	Ranges between 91 and 100%	Results indicate that atmospheric circulation of trace metals exists in Antarctica, and the various research stations may be a potential source	[79]
Fruticose and foliose lichens	Coal mine area, Figueira City, Brazil	Radio elements ^234^U, ^235^U, ^238^U, ^230^Th, ^232^Th and ^210^Po	Variable	Fruticose lichens exhibited lower polonium content than the foliose lichens sampled in the same site	[80]
*Parmotrema arnoldii*	Neotropical Andean City, Ecuador	Heavy metals in the air	Variable	*P. arnoldii* had airborne metal concentration in the order: Zn > Mn > Pb > Cd > Cu, suggesting a strong potential for monitoring air quality	[81]
*Usnea* and *Protousnea species*	Argentina-Chile borderline	Mercury and other related metals	Variable	Lichen multi-element concentration showed similar patterns across sample locations near the volcano areas	[82]
*Parmotrema* spp.	Atlantic island of São Miguel, Azores	Nitrogen and toxic heavy metals	Variable	Bioaccumulation levels were generally low to moderate in agriculture land use lichens and moderate to high in artificial land use lichens, including toxic heavy metals	[83]
*Parmotrema arnoldii*	Loja city, Ecuador	Heavy metals	-	Results confirmed that passive monitoring using lichens can be an efficient tool for evaluating heavy-metal deposition associated with urbanization	[84]
*Usnea barbata*	South Patagonia, Argentina	Iridium, platinum, and rhodium	Ir, <0.010–1.011; Pt, 0.016–2.734; and Rh, 0.063–1.298 ngg^−1^	Values detected are more likely influenced by the long-range atmospheric transport of these pollutants	[85]
*Nephroma antarcticum*, *Pseudocyphellaria granulata*, *Menegazzia magellanica*, *Parmelia sulcata*, *Chrysothrix candelaris*	southernmost Chile	UV radiation	-	UV radiation was likely an important stressor for some lichen species	[86]
*Usnea* spp.	Nahuel Huapi National Park, Patagonia, Argentina	Mercury and other related metals	Variable	Mercury contents of lichens sampled from urban and periurban sites of Bariloche city, and from undisturbed regions, demonstrate that the atmosphere of Bariloche city is enriched in mercury compared to the surroundings	[87]
Lichens	Deciduous and coniferous forests, Germany	Nitrogen	Varying levels of nitrogen enrichments	In coniferous and deciduous forests on acid soils, species with high nitrogen demand and high shade tolerance species richness increased, whereas those typical for more infertile and open forest sites decreased	[88]
*Dolichousnea longissima*, *Hypogymnia flavida*, *Sulcaria sulcata*, and *Dendriscosticta hookeri*	Near pig stock farm, central Italy	Ammonia (NH_3_) and nitrogen (N)	Varying effects of ammonia (NH3) emissions and nitrogen deposition	While *P. grisea* was the best indicator species for NH_3_ pollution, total N accumulated in *X. parietina* and *F. caperata* correlated with NH3 concentrations in them	[89]

### 2.2. Sensitivity of Lichens to Gaseous Pollutants

Lichens exhibit pronounced sensitivity to atmospheric pollution, especially gaseous pollutants, due to the absence of protective cuticle and root systems, compelling them to rely on atmospheric nutrients and water uptake for growth [22,90]. As such, direct exposure renders lichens increasingly susceptible to toxic gases, resulting in observable damage, metabolic disruptions, and potential local extinction in contaminated environments [55,59]. The extreme physiological and biochemical sensitivity of lichens to environmental pollutants substantiates their utility as bioindicators for assessing air quality [11,36,63]. For instance, a previous study indicates that when sulfur dioxide dissolves to form sulfite and bisulfite ions, it significantly disrupts lichen enzymatic activity and chlorophyll functionality [42]. Also, nitrogen oxides are found to contribute to soil acidification and eutrophication, altering lichen metabolism [32,91]. At the same time, ozone induces the formation of reactive oxygen species, resulting in oxidative stress that damages cellular lipids and proteins [92,93]. This oxidative stress leads to diminished carbohydrate production, thereby severely disrupting both the photobiont and mycobiont [94]. Physiologically, chronic exposure of lichens to these contaminants may induce chlorosis and necrosis, resulting in thallus bleaching or browning [92]. Indeed, gaseous pollutants compromise the integrity of the lichen cell membrane, thereby increasing permeability and causing electrolyte leakage [64,65]. These physiological and biochemical disturbances are likely irreversible, suggesting that pollution-related damage may ultimately lead to mortality and the potential extinction of crucial lichen species and their ecological functions. However, particular lichen species are reported to derive benefit from low nitrogen deposition for their nitrogen requirements [66,95,96], although excessive NO_x_ formation can induce nitrogen saturation and disturb their nutrient equilibrium. Such uniqueness has been observed in nitrophilous species such as *Xanthoria parietina*, which are reported to thrive in nitrogen-abundant environments [97].

### 2.3. Lichens as Bioindicators and Biomonitors of Organic Pollutants

#### 2.3.1. Persistent Organic Pollutants

Persistent organic pollutants (POPs) are a group of hazardous, bioaccumulative chemical substances characterized by their long lifespan in environmental matrices [10,44,98]. Lichens exhibit distinct attributes that are suitable for monitoring the spatial distribution and accumulation patterns of POPs within ecosystems [44,56]. Lichens are well-suited for such monitoring programs owing to their ability to absorb airborne contaminants, as discussed earlier. The mycobiont component of lichens, comprising fungal hyphae, demonstrates a propensity to sequester high concentrations of lipophilic POPs due to the presence of fatty tissues [99]. Furthermore, the characteristic slow growth rates and lichens’ long lifespans enable integration and continuous exposure to pollutants over extended temporal scales. In the absence of efficient excretory mechanisms, the accumulated POPs persist within the lichen thalli, thereby forming stratified records of environmental contamination [10,100,101]. Lichens facilitate the accumulation of POPs via multiple pathways: (1) Atmospheric deposition, wherein gaseous and particle-bound POPs adhere to lichen surfaces and infiltrate the hyphal matrix, (2) Hydrological inputs, such as precipitation and snowmelt, which convey dissolved POPs to lichen assemblies, and (3) The complex surface morphology of lichens, which effectively captures and retains airborne particulates laden with adsorbed POPs. Following incorporation into lichen tissues, non-polar POPs preferentially localize within fungal cellular membranes and lipid bodies. Additionally, certain chlorinated compounds are known to form complexes with cellular proteins [102], while particulate matter undergoes physical entrapment within the hyphal network of the lichen.

#### 2.3.2. Lichens as Microplastic and Nanoparticle Pollution Monitoring

Lichens have emerged as highly effective biomonitors for detecting two prominent emerging contaminants: microplastics and nanoplastics [24,25,26,27,103]. These pollutants are characterized by their diminutive size, which significantly influences their ecological impact [10,11,36,44,58,102,104,105]. The unique morphological and physiological traits of lichens render them particularly adept at monitoring these microscopic pollutants [10,11,38,104]. As synthetic particulates become increasingly pervasive within ecosystems, lichens provide a cost-effective and biologically pertinent system for tracking their distribution. Microplastics and nanoparticles challenge conventional pollution-monitoring methods due to their small size, heterogeneous chemical composition, and complex environmental interactions.

Lichens employ diverse mechanisms for particle capture and retention via multiple physical and physiological processes: (1) The complex surface topography of foliose and fruticose lichens functions as a three-dimensional mesh, effectively capturing airborne particulates [25,26,27,68]. Hyphal projections and cortical structures in species such as *Hypogymnia physodes* can entrap particles as small as 50 nm. (2) The charged surfaces of lichen cell walls, in conjunction with synthetic particles, facilitate adhesion, a phenomenon particularly evident with nanoparticles [25,26,27]. This effect is notably enhanced under arid conditions, when lichen surfaces exhibit higher electrostatic charge. (3) The waxy upper cortex of numerous lichen species selectively attracts hydrophobic microplastics, such as polyethylene and polypropylene, resulting in a concentration effect. (4) Certain nanoparticles infiltrate lichen thalli through pores within the fungal hyphae, subsequently becoming integrated into cellular structures.

### 2.4. Oxidative Stress Biomarkers in Lichens

Lichens, when subjected to frequent environmental stressors, exhibit potential oxidative stress and cellular damage, prompting the use of a diverse array of biomarkers to indicate stress levels and adaptive responses [10,37,44,106]. These biomarkers comprise enzymatic antioxidants, non-enzymatic antioxidants, oxidative damage byproducts, and stress-induced secondary metabolites [107,108]. It is crucial to elucidate that the investigation of lichen biomarkers provides significant insights into their physiological health, pollution tolerance, and ecological resilience against oxidative stressors [65], originating from both natural and anthropogenic origins. A notable example is desiccation-rehydration cycles, prevalent in lichens, which can induce metabolic disturbances [109]. Additionally, exposure to ultraviolet radiation leads to the production of reactive oxygen species due to photosynthetic inefficiency. As a regulatory adaptation, lichens synthesize distinctive secondary metabolites, which serve as a rich source of bioactive compounds with antioxidant properties [107,108]. Notably, bioactive compounds such as usnic acid, parietin, and atranorin detoxify the lichen system by eliminating toxic compounds, thereby serving as a biochemical adaptation to oxidative stress [107,110,111]. Monitoring these metabolites yields critical information on lichens’ stress response and prevailing environmental conditions [110,111].

### 2.5. Gradient Analysis of Lichens: A Powerful Tool for Environmental Assessment

Gradient analysis constitutes a methodological framework that facilitates a rigorous examination of changes in lichen communities along environmental gradients. This analytical technique has been identified as one of the most effective methodologies for monitoring the ecological responses of lichens [112,113]. The gradient analysis approach is particularly critical for evaluating the impacts of air pollution, climate change, habitat fragmentation, and other ecological stressors [114]. By examining lichen distribution patterns across gradients, significant thresholds in environmental pollution impacts have been identified, enhancing predictions of ecosystem responses to prospective disturbances. In industrial areas, pollution gradients can be characterized by several key environmental stressors impacting lichen populations. Elevated levels of air pollutants are known to diminish lichen species richness and diversity substantially [39,94,114,115].

Furthermore, industrial activities such as mining, construction, and energy production release heavy metals into the atmosphere, which are absorbed by lichens [55,56,57]. The bioaccumulation of these metals can adversely affect lichen health, as evidenced by decreased photosynthetic activity, reduced growth, and tissue damage [41,42]. Observational data indicate that lichens located proximal to industrial sites frequently exhibit a pronounced gradient of declining diversity [57,116]. Consequently, lichen communities in these zones are often sparse or predominated by a limited number of pollution-tolerant species, such as *Xanthoria parietina*, known for its resilience to air pollutants [41]. Conversely, as one moves away from pollution sources, species diversity tends to increase, with a more noticeable presence of sensitive species such as *Lobaria pulmonaria* [19,106]. These species are typically indigenous to environments with cleaner air and exhibit heightened sensitivity to pollutants such as sulfur dioxide and heavy metals [19].

## 3. Long-Term Biomonitoring of Lichens: Sentinels of Environmental Change

Lichens serve as natural bioindicators of air quality, climate change, and ecosystem stability, owing to their distinctive physiological characteristics, primarily the absence of root systems, atmospheric nutrient assimilation, and heightened sensitivity to contaminants [3,5]. Lichen-based biomonitoring generates integrative, biologically meaningful datasets that reflect the cumulative exposure of ecosystems to environmental stressors. The majority of lichen species exhibit slow growth rates and long lifespans, enabling them to accumulate atmospheric and edaphic pollutants while colonizing a wide range of substrates, including tree bark, rock, and soil [3,4,37,42]. The absence of a protective cuticle facilitates the direct absorption of atmospheric nutrients, thereby rendering them exceptionally responsive to air pollutants, such as sulfur dioxide, nitrogen oxides, and heavy metals [15,32,59,117]. Nonetheless, it has been demonstrated that lichen species exhibit differential tolerance levels to pollution, enabling the application of community composition as a bioindicator of environmental stress [6,13,40,115]. These characteristics underscore the usefulness of lichens as a vital biological tool for detecting gradual environmental changes that may not be readily apparent through conventional assessment methods [37]. Long-term lichen biomonitoring involves systematically repeating surveys of lichen communities, providing an opportunity to monitor shifts in species composition attributable to pollution, climate change, or habitat modification. For instance, the decline in pollution-sensitive species such as *Lobaria pulmonaria* (a sensitive epiphytic lichen) in industrialized regions has been correlated with historical sulfur dioxide emissions [118,119]. Furthermore, the proliferation of nitrophilous species like *Xanthoria parietina* in urban environments reflects increased nitrogen deposition from vehicular and agricultural sources [120].

### 3.1. Lichens in Climate Change Monitoring: Sentinels of a Warming World

In response to the accelerating global climate change, researchers are increasingly utilizing eco-friendly natural indicators to monitor environmental changes. Among these biological indicators, lichens have proven particularly effective for monitoring climatic variations [36,63,118,121,122]. Their unique biological characteristics, widespread distribution, and pronounced sensitivity to environmental shifts make them particularly well-suited as model organisms for investigating the multifaceted effects of climate change [33,40,59,123]. Lichen species exhibit specific environmental preferences [97], with some thriving in arid environments while others require constant moisture [124]. This ecological specialization allows lichen communities to serve as precise indicators of microclimatic conditions. For example, the presence or absence of specific lichen species in lacustrine sediments can reflect historical climatic conditions [36]. Lichen communities are providing unequivocal evidence of ongoing global environmental change across diverse ecosystems (Table 2).

In Arctic and alpine ecosystems, rising ambient temperatures are inducing measurable shifts in the spatial distribution patterns of lichen communities [147,148,149]. Despite these temperature effects, a recent study in Antarctica found that *Usnea aurantiaco-atra* showed no significant effect of long-term warming; however, *Cladonia* species were most affected by water-stress conditions [135]. This suggests that some Antarctic lichens tolerate high temperatures better than desiccation, and that while climate change impacts may be species-specific [54], effects associated with decreased water availability may harm lichen communities in these ecosystems [142]. As such, some species previously found at lower elevations are progressively migrating upward, while cold-adapted communities are declining [149]. In arid regions, however, severe drought has reduced the vitality and coverage of desert crust lichens, which play pivotal roles in soil stabilization [147,148]. For example, a long-term annual observational dataset indicates a consistent decline in N-fixing lichen cover (dominated by *Collema* species) from 1996 to 2002, coinciding with a period of extended drought [150]. As a result, the *Collema* communities never recovered from the consequences of this drought. However, increased humidity in some temperate ecosystems has facilitated the expansion of moisture-dependent species, such as *Lobaria pulmonaria*, into novel habitats [125].

As integral components of global carbon cycles, lichens contribute to climate change monitoring in dual capacities. Mat-forming lichens, such as *Cladonia* species, serve as significant carbon sinks [126,151], as their slow decomposition rates result in long-term carbon sequestration. Climate-induced changes in these lichen-dominated ecosystems could thus have profound implications for global carbon budgets [151]. Furthermore, lichens associated with cyanobacterial symbionts contribute to nitrogen fixation, thereby influencing nutrient cycling in highly sensitive ecosystems [15,152]. Changes in their abundance due to climatic shifts may affect primary ecosystem processes, with cascading effects on vegetation patterns and carbon sequestration capacities [15,152].

### 3.2. Lichens as Biodiversity and Habitat Assessment: Nature’s Ecological Barometers

Lichens function as precise bioindicators of ecological integrity across a range of ecosystems [37,38,42,44]. They exhibit unique physiological and morphological traits that render them essential for assessing ecosystem health, detecting environmental alterations, and informing conservation strategies [10,37,38,44]. As previously mentioned, their slow growth rates facilitate the accumulation of atmospheric contaminants and the documentation of ecological shifts over extended periods. Lichens inhabit almost all terrestrial ecosystems, thereby enabling fundamental biodiversity evaluations across various biogeographic zones [11]. Consequently, forest ecosystems constitute a critical application area for lichen-based biodiversity assessments. Lichen assemblages vary between pristine old-growth forests and managed woodlands, making them exceptional indicators for monitoring ecosystem quality. Notably, some lichen species, such as *Lobaria pulmonaria*, are susceptible to changes in forest microclimates and serve as flagship species for the conservation of old-growth forests [38,124].

The presence of lichens incorporating nitrogen-fixing cyanobacteria indicates high-quality forest habitats characterized by stable microclimates and minimal air pollution [152,153]. These lichen species play a crucial role in forest nutrient cycling and act as bioindicators of ecosystem health [32,142]. Consequently, a decline in these species often signifies a loss of biodiversity within forest ecosystems [142]. Furthermore, the diversity of epiphytic lichens has become a pivotal aspect of forest certification programs, as rich lichen communities are frequently associated with ecosystem stability [19,106,154]. In assessing agricultural and urban ecosystems, lichens provide essential insights into habitat fragmentation and environmental stress [155]. Urban regions generally exhibit lower lichen diversity than natural environments, and sensitive species are rapidly disappearing. The composition of urban lichen communities mirrors the extent of anthropogenic activities, with nitrophilous species such as *Xanthoria parietina* prevailing in polluted urban centers [3,16,106,147,148,155]. Lichens are effective tools for monitoring habitat connectivity in fragmented ecosystems. The presence of species with limited dispersal capabilities in urban parks or along tree corridors indicates the presence of functional ecological networks, whereas their absence underscores barriers to species movement [156]. In agricultural contexts, lichen communities on boundary trees and hedgerows can reveal the ecological impacts of varying farming practices [157,158], with organic farms generally supporting more diverse lichen assemblages than conventional monocultures [159].

## 4. Lichens’ Response to Major Global Change Factors

### 4.1. Lichens and Climate Change

#### 4.1.1. Interactions with Temperature and Precipitation Shifts

**Figure 3:**
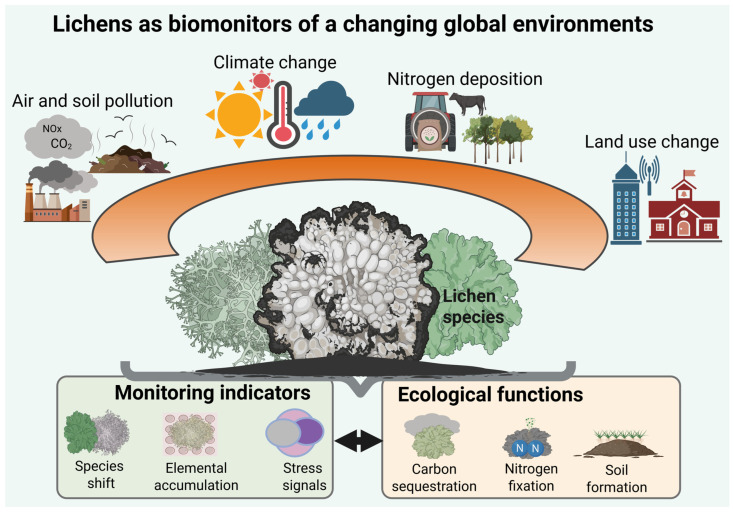
Conceptual visualization of roles of lichens as biomonitors in a changing global environment.

Lichens exhibit a pronounced sensitivity to environmental perturbations, which considerably affects their growth, geographic distribution, and overall vitality [112,114,127]. Indeed, changes in temperature and precipitation patterns significantly influence the physiological mechanisms and ecological functions of lichens [160]. As previously indicated, lichens possess the remarkable ability to endure extreme habitats such as the Arctic, desert regions, and high-altitude environments [7,54,161]. Nevertheless, notwithstanding their resilience, lichens remain susceptible to temperature and precipitation fluctuations due to their dependence on moisture availability for photosynthetic activity [5,148,160]. Consequently, alterations in these climatic variables can detrimentally impact their metabolic functions, reproductive success, and growth dynamics. Specifically, elevated temperatures may disrupt physiological processes by reducing the moisture content of the lichen thallus, or the lichen body [162]. In warmer climates, lichens can suffer from reduced moisture availability due to increased evaporation rates, leading to physiological stress and an inability to perform photosynthesis effectively [148,162]. This phenomenon is exemplified by the lichen species *Cladonia*, which is prevalent in high-latitude regions such as the Arctic [39,40,163]. Specifically, increasing Arctic temperatures have substantially altered the distribution of *Cladonia* species [161]. The warming climate has also contributed to the decline of certain lichen species, reducing their abundance and diversity [135] (Figure 3).

Elevated temperature regimes may facilitate the expansion of species typically restricted to cooler environments into warmer habitats [7,161,164]. The yellow–orange lichen *Xanthoria parietina* has been observed to thrive in elevated, warmer climates [7,164]. In Europe, *X. parietina* has migrated into urban areas, presumably due to urban heat, thereby illustrating how certain lichen species may benefit from increased temperatures, particularly those adapted to heat [135,164,165]. Furthermore, precipitation constitutes one of the primary factors influencing lichen growth and distribution [142]. Lichens assimilate moisture from the atmosphere and their immediate environment [119], rendering them particularly susceptible to variations in precipitation patterns. For instance, in Mediterranean climates, where summer droughts are intensifying due to climate change [166,167], lichen species such as *Ramalina* and *Lecanora* are encountering substantial challenges [160,168,169]. Thus, a reduction in precipitation is directly correlated with lichens, as these organisms depend on consistent rainfall for population growth [155]. Similarly, in arid desert regions, alterations in rainfall patterns can impact the abundance of lichen species resilient to drought [7,164,168]. A recent investigation has found that rising temperatures, coupled with declining precipitation, have led to the contraction of certain lichen populations and the proliferation of others, notably those adapted to arid conditions [130].

Contrary to some expectations, certain lichen species exhibit a strong dependency on elevated moisture levels to sustain their photosynthetic mechanisms, with increased humidity providing an optimal growth environment [150]. Consequently, the interplay between changes in temperature and precipitation patterns can significantly affect lichen community dynamics [3,5,150]. For example, in ecosystems experiencing simultaneous increases in temperature and precipitation, there is a potential for the proliferation of lichen species, favoring moist habitats. In contrast, those adapted to arid conditions may experience a decline [127]. Conversely, a scenario of rising temperatures coupled with decreased precipitation is likely to lead to a reduction in overall lichen biodiversity, as numerous species may struggle to adapt to the dual pressures of elevated temperatures and limited moisture [125,127].

#### 4.1.2. Lichens as an Indicator of Synergistic Stressors: Unveiling Complex Environmental Challenges

Lichens serve as a sensitive bioindicator of multiple interacting stressors, responding to the synergistic effects of various global change stressors [39,95,125,127,147,170]. They can reveal the complex interactions between factors such as air pollution, climate change, and habitat fragmentation, resulting in ecological impacts that exceed the sum of the individual stressors. Synergistic stressors occur when two or more environmental factors interact, producing effects more severe than their individual impacts. Due to their direct atmospheric exposure and absence of protective tissues, lichens are particularly susceptible to these interaction effects [39,95,125,127,147,170]. For instance, while moderate air pollution might reduce lichen vitality, its combination with drought stress induced by increased temperatures can rapidly disrupt lichen communities. The preference of many epiphytic lichens for specific bark pH levels renders them sensitive to the combined effects of acid rain and nutrient deposition [171,172], where neither stressor alone would induce such dramatic changes.

One extensively studied synergistic effect involves the interaction between atmospheric pollutants and climate variables: (1) Elevated temperatures amplify ozone damage to lichen membranes, with *Flavoparmelia caperata* exhibiting 40% more cellular damage at 30 °C compared to 20 °C under identical ozone concentrations [35,122,127]. (2) Lichens enriched with nitrogen (*Xanthoria* spp.) demonstrate increased drought sensitivity due to altered water-holding capacity in their thalli, with field studies indicating that nitrogen-loaded specimens desiccate more rapidly during dry periods [151]. (3) Metal-contaminated lichens (*Hypogymnia physodes*) exhibit decreased synthesis of UV-protective compounds [43,66], heightening their vulnerability to radiation damage in high-altitude, ozone-depleted environments. (4) The edge effects in fragmented forests expose sensitive *Lobaria* species to compounded microclimate changes, leading to 60% higher mortality in small forest patches during heatwaves [173,174]. (5) Urban lichens are subject to simultaneous heat stress and particulate matter deposition, resulting in a reduction in photosynthetic capacity by up to 70% compared to rural populations [67,104,175].

Lichens exhibit physiological responses to combined stressors via complex, interconnected pathways. Specifically, (1) concurrent exposure to heavy metals and oxidative stress induced by ozone results in the depletion of antioxidant defenses in lichens, such as glutathione and superoxide dismutase [176,177,178]. This depletion occurs at relatively low pollutant concentrations, leading to cellular damage. (2) *Peltigera* species experience altered water relations due to nitrogen enrichment [32,179], rendering them more susceptible to variations in precipitation—a significant concern for ecosystems in Mediterranean climates. (3) Elevated atmospheric CO_2_ concentrations differentially impact the algal and fungal components of lichens [147], potentially destabilizing the symbiotic relationship, particularly when additional stressors such as heavy metals or acidification are present. (4) Under extreme UV radiation and temperature, lichen employs secondary products, including melanin and parietin, which act as a protective shield or “sunscreen,” which physically screens out or filters the harmful UV-B and UV-A radiations [180,181], ultimately preventing any oxidative damage to the photobiont’s DNA and photosynthetic apparatus. For example, a recent study observed a high degree of photoinhibition resistance in the Antarctic lichen *Xanthoria elegans* in a short, high-light stress manipulation study [182]. Additionally, usnic acid, a dynamic multifunctional secondary metabolite also plays a central role in the adaptive strategy of lichens inhabiting environmentally harsh, high-irradiance habitats [183]. In addition to functioning as an intrinsic photoprotective “sunscreen” compound, it undergoes context-dependent modulation in response to both ultraviolet radiation and thermal stress [184], thereby exemplifying the remarkable phenotypic plasticity and environmental responsiveness of the lichen symbiosis. Also, hydrophobins play critical roles in waterlogging conditions by facilitating gas exchange, thereby preventing the photobiont from drowning [185,186]. Thus, these fungal proteins assemble into a hydrophobic or waterproof layer on the cell wall surface, keeping the interhyphal space dry and open for CO_2_ and oxygen diffusion.

### 4.2. Urbanization and Habitat Fragmentation

#### 4.2.1. The Rise in Lichen Deserts

The accelerated urbanization process has led to significant ecological disturbances, notably resulting in the decline of lichen populations [104,150,187]. Lichens, being sensitive bioindicators of environmental health, are particularly susceptible to the impacts of urbanization and habitat fragmentation [27,188]. The phenomenon known as the “lichen desert,” associated with the deposition of acid rain from industrial effluents and characterized by minimal lichen presence, offers compelling evidence of anthropogenic alterations to ecosystems [104,189]. These desolate areas can extend 10–50 km from urban centers [104,158], exhibiting a predictable succession pattern, starting with the disappearance of sensitive species such as *Lobaria pulmonaria*, followed by nitrogen-sensitive varieties, until only the most pollution-tolerant species, such as *Lecanora conizaeoides* [25,26,27,104]. Thus, *L. conizaeoides* is tolerant of acid rain but highly sensitive to nitrogen, and is now becoming rare with increasing atmospheric nitrogen deposition.

Urban environmental modification introduces multiple stressors to lichen survival. For instance, (1) emissions from vehicles and industrial activities contribute to excessive nitrogen deposition [179], which favors the proliferation of nitrophilous species like *Xanthoria parietina*, while leading to the elimination of nitrogen-sensitive lichens [16,97,190]. For instance, a dataset of 286 epiphytic lichens observed on 1155 trees across 83 ForestBIOTA plots in Europe indicates that nitrogen deposition accounted for 56.7% of the variation in microlichen abundance [191]. Notably, the reactive forms of nitrogen (i.e., ammonia, NH_3,_ and nitrogen oxide, NO_x_) have also been shown to induce profound inhibitory effects on the sensitive physiological balance of lichen. Especially, NO_x_, a key pollutant from vehicular traffic, can directly damage lichens’ cellular processes [192]. Thus, the differential response of two acidophytic lichens (*Cladonia* and *Usnea*) to increased reactive nitrogen availability has recently been documented [193]. (2) Particulate matter such as PM2.5 and PM10 adheres to lichen surfaces, impeding light penetration and gas exchange. Populations of *Parmelia saxatilis* in high-PM zones of Beijing demonstrate a 60% reduction in photosynthetic rates [67,194]. (3) Urban lichens accumulate heavy metals like lead, zinc, and copper at concentrations 10–100 times greater than rural counterparts, disrupting enzymatic processes [195]. *Hypogymnia physodes* in industrial zones exhibit severe membrane damage when metal concentrations exceed 50 μg/g. (4) Ground-level ozone exposure results in chlorophyll degradation, with *Flavoparmelia caperata* displaying a 40% decrease in chlorophyll content in high-ozone areas of New Jersey [69,196].

#### 4.2.2. Urbanization and Habitat Fragmentation: The Impacts of Edge Effects on Lichen Communities

The delineation between developed and natural environments is referred to as the habitat edge [197], establishing a distinct zone characterized by ecological disturbances. The microclimatic, chemical, and biological alterations occurring at these habitat edges exert significant influences on lichen communities [197,198]. Consequently, habitat edges induce three primary effects on lichens: A pronounced gradient exists between urban communities and vegetation, resulting in substantial variations in temperature, humidity, and wind exposure [199]. For instance, research conducted in Chicago parks indicates that trees situated at edges experience summer temperatures elevated by 3.2 °C and reduced humidity compared to those in interior forest stands [200], thereby diminishing moisture availability for sensitive lichens such as *Peltigera* species [201,202]. Furthermore, observations within Berlin’s Grunewald Forest reveal nitrogen deposition levels that are quintuple those found in forest interiors at edges [203], prompting the replacement of acidophilic *Usnea* species with nitrophilous *Xanthoria* communities within 50 m from the edge [197,204].

Additionally, edge habitats are predominantly populated by weedy lichen species and pathogenic fungi. Analyses of *Lobaria pulmonaria* show that edge populations harbor a higher prevalence of fungal pathogens than interior groups [205], thereby reducing reproductive success. The edge effects engendered by urban habitat fragmentation signify a significant reorganization of ecological conditions affecting lichen communities [3,5,174,197]. These boundary zones function as filters, selectively eliminating sensitive species while favoring generalists, leading to biotic homogenization across urban environments. Nonetheless, lichens exhibit notable resilience, as certain species develop thicker cortices, altered reproductive strategies, and enhanced pollution tolerance at edges [3,5,174,197]. Preserving lichen biodiversity in urbanizing areas necessitates the design of green spaces that minimize edge impacts while maintaining functional connectivity [206,207]. As urban expansion continues, understanding and mitigating edge effects is imperative for conserving these crucial components of urban ecosystems [67,187,198].

### 4.3. The Impact of Biological Invasion on Lichens as Biomonitors of Global Change

Biological invasion refers to the introduction and subsequent proliferation of non-indigenous species within ecosystems [208,209]. The introduction of these invasive species poses a substantial threat to lichen communities, potentially undermining their effectiveness as bioindicators of pollution [210]. Invasive flora and fungal species can outcompete native lichens for spatial and resource niches. For instance, aggressive invasive plants such as *Bromus tectorum* and *Lantana camara* modify microhabitats by altering light penetration, moisture availability, and soil chemical and physical properties [210,211]. Given that lichens depend on specific environmental conditions for survival, such modifications can accelerate their decline or even local extinction. Moreover, invasive fungi or microbial entities may parasitize lichens directly, further diminishing their populations [212]. A reduction in lichen diversity and abundance could compromise their utility as indicators of pollution, as fewer species remain to signal environmental variations.

Biological invasions can alter nutrient cycling and soil chemistry, thereby indirectly affecting the vitality of lichen communities [213]. For example, invasive nitrogen-fixing flora, such as *Acacia* spp., may elevate soil nitrogen concentrations [214], potentially promoting the proliferation of pollution-resistant lichen species at the expense of more sensitive counterparts. Given that air quality monitoring requires the presence of both sensitive and resistant lichen species to accurately assess atmospheric conditions, shifts in species composition could compromise their ecological roles. Specific invasive taxa may serve as conduits for pollutants or pathogens detrimental to lichen health [215,216], while others could emit allelopathic substances that inhibit lichen growth [217,218]. Additionally, invasive microorganisms may introduce novel pathogens, diminishing or eradicating lichen populations [34,215], thus reducing their reliability for longitudinal pollution monitoring. Furthermore, lichens are integral to standardized methods of air quality evaluation, such as the Index of Atmospheric Purity, which depends on species diversity and abundance metrics [209,219,220]. Biological invasions can disrupt these indices by leading to the extirpation of sensitive lichen species, potentially underestimating pollution levels.

## 5. Implications of Lichen-Based Biomonitoring of Global Environmental Changes

The importance of utilizing lichens as bioindicators of global environmental change lies in their capacity to serve as an eco-friendly tool for assessing and managing ecosystem condition at continental scales. By developing standardized monitoring protocols, the scientific community can now directly compare the concurrent effects of major anthropogenic drivers, such as reactive nitrogen enrichment, airborne microplastics, heavy metals, and climate change, on ecosystems across Europe, North and South America, Asia, and other parts of the world. Additionally, lichen-based monitoring has demonstrated that lichens function not only as passive indicators of environmental quality but also as integral components of ecosystem processes, regulating nutrient fluxes and hydrological dynamics. The decline of lichen communities under multiple, interacting stressors, including synergistic effects between climate change and atmospheric pollution, indicates the potential for substantial changes in vegetation structure, ecosystem processes, and associated biodiversity. Finally, lichen-derived bioindicators provide a quantitative basis for modeling future environmental trajectories and for designing evidence-based conservation and management strategies to enhance the resilience of vulnerable ecosystems.

## 6. Conclusions

Lichens, which are complex symbiotic organisms constituted by fungi in association with photosynthetic algae or cyanobacteria, serve as crucial yet often underestimated indicators of environmental health. Their exceptional adaptability enables them to thrive in a wide range of ecosystems, from isolated mountain summits to urban environments. However, their lack of protective cuticles and their reliance on atmospheric absorption render them particularly susceptible to air pollution. These dual characteristics categorize lichens as invaluable bioindicators, capable of indicating alterations in air quality through physiological stress responses, shifts in species composition, and biodiversity loss. From an ecological perspective, lichens are integral to primary succession, soil formation, and nutrient cycling, particularly via nitrogen fixation, while providing essential food and habitat resources for diverse wildlife. Nonetheless, increasing pollution from industrial emissions, vehicular exhaust, agricultural activities, and biomass combustion poses a significant threat to their survival. These impacts are exacerbated by climate change, including rising temperatures, altered precipitation patterns, and habitat modifications, creating compounded stressors that hasten lichen decline. Progress in biomonitoring has evolved lichen-based assessments from rudimentary presence–absence surveys to sophisticated analyses encompassing bioaccumulation studies, biodiversity metrics, and remote sensing [143,221,222,223] (see also Table S1). Notably, epiphytic lichens effectively delineate pollution gradients and chronicle long-term trends in air quality. Emerging methodologies in molecular biology and metabolomics further augment their utility, enabling the detection of subtle pollutant impacts and species-specific responses. This review synthesizes current insights into lichen ecology, their mechanisms of pollution sensitivity, and their expanding role in environmental monitoring. As air quality emerges as an increasingly critical global concern, lichens highlight a profound truth: the most inconspicuous organisms often provide the most revealing insights into the condition of global ecosystems.

## Figures and Tables

**Table 2:** Evidence of global change effects on lichen communities.

Species	Location/Region	Observed Range	Effects/Results	References
*Flavocetraria nivalis* and *Cladonia mitis*	Svalbard Archipelago	–7.2 °C to +0.3 °C	*F. nivalis* exhibited a bioaccumulation factor (BAF) > 1 for a greater number of metals compared to *Cl*. *mitis*, indicating higher uptake potential	[62]
*Evernia mesomorpha*	Marcell Experimental Forest in north-central Minnesota	Above +2 °C	The substantial negative impacts of warming and/or lower humidity on *E. mesomorpha* were driven by a loss of photobiont activity	[122]
*Cladonia mitis*, *Cetraria islandica*, and *Nephromopsis nivalis*	High mountain plateau in central Norway	Mean annual temperature of −1.1 °C	The lichen’s stronger tolerance against thaw–freeze and ice encasement than co-existing plants opposes the general effects of summer climate warming, where lichens may succumb under greater plant growth and warmer soils	[125]
Lichens	Mount Sanddalsnuten	Mean July temperature of 7.0 °C, a mean January temperature of −10.7 °C	Lichen-specific thallus area and water holding capacity were unresponsive	[126]
*Diploschistes diacapsis*, *Squamarina lentigera*, *Fulgensia subbracteata*, and *Buellia zoharyi*	Aranjuez Experimental Station, central Spain	Mean annual temperature and rainfall values of 15 °C	Over time, lichens colonized the LIBC plots, but this process was hampered by warming	[127]
*Usnea aurantiaco-atra*	Antarctic Tundra	1.1 to 2.6 °C and 2.6 to 4.8 °C	Temperature is highlighted as the main driver for projections, and thus climate warming will lead to an average increase in net primary photosynthesis of 167–171% at the end of the century	[128]
*Cetraria cucullata*, *Cladina mitis*	Low Arctic Canada	−38 ± 0.7 °C in January to a maximum of 20 ± 0.4 °C in July	Climate warming could reduce cryptogam plant community stability in the low Arctic tundra	[129]
*Flavocetraria cucullata*, *Cetrariella delisei*, *Flavocetraria nivalis*, *Cladina arbuscula*, *Cladonia unicalis*, *Ochrolechia frigida*	Latnjajaure field station	Mean annual air temperature ranged from −0.76 to −2.92 °C between 1993 and 2013	The results from the long-term warming study imply that arctic and high alpine lichen communities are likely to be negatively affected by climate change and an increase in plant canopy cover	[130]
*Cetraria aculeata*	Seminatural sandy grassland in Klucze village, Poland	The critical temperature for metabolism is around 35 °C	Heavy rainfall during high temperatures may significantly deteriorate the physiological condition of melanized thalli	[131]
Lichens	Swedish subarctic birch forest and subarctic/subalpine tundra to Alaskan arctic tussock tundra	From early spring (after snow melt) to autumn	Natural gradient studies and experimental studies show that cryptogam diversity and abundance, especially within lichens, are likely to decrease under arctic climate warming	[132]
*Ochrolechia*, *Cetrelia*, *Cladonia*, *Lepraria*, and *Micarea* sp.	Białowieża Forest	Mean annual air temperature by 1.1 °C (from 6.3 to 7.4 °C)	Twenty-five species demonstrated a shift to co-occur with lichens of higher nitrogen demands, 15 demonstrated higher light demands, 14 demonstrated higher temperature preferences, and six demonstrated lower moisture preferences	[133]
*Xanthoparmelia austroafricana*, X. *hyporhytida*, *Xanthomaculina hottentotta*	Arid South African ecosystem	Artificially elevating temperatures (increase 2.1–3.8 °C)	Climate warming, interacting with reduced precipitation, will negatively affect carbon balances in endemic lichens by increasing desiccation damage and reducing photosynthetic activity time	[134]
*Himantormia lugubris*, *Usnea aurantiacoatra*, *Cladonia borealis*	Antarctic tundra	15.9 °C	Endemic *H. lugubris* showed the strongest effect of long-term warming on primary photochemistry	[135]
*Psora decipiens*	Climate Change Outdoor Laboratory of Rey Juan Carlos University	~2.3 °C	The functional response observed could limit the growth and cover of biocrust-forming lichens in drylands in the long term, negatively impacting key soil attributes such as biogeochemical cycles	[136]
Fruticose lichens	Toolik Lake Research Station, Alaska	Daytime air temperature by 1–5 °C during the snow-free period	The proportion of several lichenized fungi decreased with warming	[137]
*Evernia mesomorpha*	Marcell Experimental Forest in northern Minnesota	Every +1 °C	Changing patterns of warming and drying would decrease or reverse *Evernia* growth at its southern range margins, with potential consequences for the maintenance of local and regional populations	[138]
*Usnea aurantiaco-atra*	Antarctic Peninsula	2.66 ± 0.12 °C and 2.70 ± 0.15 °C	Results show that *U. aurantiaco-atra* performed better at the summit, but desiccation had a significant negative impact on the species at the lowest site, confirming water relations are the main drivers of biodiversity change along the altitudinal gradient	[139]
*Cladonia* and *Parmotrema* species	mid-Atlantic Coast of eastern North America	Variable effect of sea-level rise	*Cladonia* species occupy significantly less than *Parmotrema* species and are predicted to lose more of their distributions to sea-level rise	[140]
*Bulbothrix laevigatula*, *Cryptothecia rubrocincta* *Leptogium cyanescens* and *Parmotrema rampoddense*	Southeast Coastal Plain, Florida	Variable effect of sea-level rise	Sites lacking salt-sensitive lichens such as *B. laevigatula*, *C. rubrocincta*, *L. cyanescens*, and *P*. *rampoddense* could indicate areas that are at high risk to sea-level rise at a fine scale	[141]
*Lobaria pulmonaria*	Natural donor population, oak–hazelwood at Loch Barnluasgan, Scotland	Variable effect of sea-level rise	Light and moisture deficit interaction reduced the net growth of *L. pulmonaria*	[142]
Lichens	Urban-rural gradient, city of Oslo	Varying effects of acid rains	Excess nitrogen and sulfur deposition alter the diversity and effective establishment of acidophytic lichens	[143]
*Dolichousnea longissima*, *Hypogymnia flavida*, *Sulcaria sulcata*, and *Dendriscosticta hookeri*	Trans-Himalayas regions	Varying effects of pH, temperature, and vapor pressure	Increased winter temperatures were most influential at the highest elevation region, whereas reduced relative humidity was most important at mid-elevations; however, increased vapor pressure deficit was beneficial to *D. longissima* but detrimental to four species	[144]
Epiphytic macrolichens	Tropical montane forest, southern Ecuador	Varying effects of deforestation and forest conversion	Total richness tended to decrease when the range of the disturbance increased, and the similarity within sites decreased when the range of the disturbance was greater	[145]
Multispecies (~1475 lichen species)	Lichens along altitudinal gradients in the Indian Himalaya regions	Varying range of temperature and precipitation	Change in precipitation and temperature significantly affected lichen diversity in both western and eastern Himalaya, potentially serving as a biomonitor of global change	[146]

## Data Availability

The data presented in this study are available in this manuscript, and constructs can be requested from the corresponding author.

## Supplementary Materials

The following supporting information can be downloaded at: https://www.mdpi.com/article/10.3390/biology15060478/s1, Table S1: Progress and methodologies for determining lichen responses to environmental change variables in the last decade.
