# Supplementary material for: Ecological Roles of Lichens as Monitors of a Changing Global Environment
Source: Biology (Basel). 2026 Mar 17;15(6):478. doi: 10.3390/biology15060478 (PMC13024471; doi:10.3390/biology15060478)
Supplement: Supplementary file 1 [file biology-15-00478-s001.zip › biology-4144230-supplementary.pdf]

Supplementary Table S1: Progress and methodologies for determining lichen responses to environmental change variables in the last decade

Global change factor	Study setting/ duration	Species	Lichen response determination method(s)	Reference
Toxic elements	Field study	*Usnea antarctica*	Laser ablation inductively coupled plasma mass spectrometry	[50]
Precipitation and light	24-month experiment	*Lobaria pulmonaria*	Predictive bioclimatic models	[116]
Microclimate conditions	Field sampling	*Usnea barbata*	Inductively coupled plasma mass spectrometry	[223]
Simulated climate change	2018-2020 Field study	*Dolichousnea longissima*	UniT loggers (UT330) and Hygrochron iButton loggers (DS1923)	[144]
Atmospheric elements	3 to 12-month field exposure experiment	*Xanthoparmelia camtschadalis*	Inductively coupled plasma mass spectrometer	[45]
18 years of natural and experimental Warming	18-year natural and field experimental	Multispecies	Field Open top chambers and measure temperatures with Delta™ and Tinytag™ loggers	[130]
Acid rains via Nitrogen and sulfur deposition	47-year field observation	Epiphytic macrolichen	Long-term continuous field observation	[224]
Anthropogenic disturbance	Field sampling	Multispecies	A habitat quality index based on an established forestry metric	142
Airborne microplastics	Field observation and chemical analysis	*Cladonia and Xanthoria*	Physical identification of fibres and micro-Fourier-transform infrared spectroscopy (micro-FTIR) for chemical feature observation	[27]
Reactive nitrogen availability	Laboratory experiment	*Cladonia and Usnea*	Photobiont and mycobiont vitality, chitin quantification, nitrogen content, and stable isotopes analysis	[195]
Sea-level rise	Field sampling	*Cladonia* and *Parmotrema* species	Field dataset for species distribution analysis	[140]
Passive warming	14-year field experiment	*Himantormia lugubris, Usnea aurantiaco-atra,* and *Cladonia aff. gracilis*	Desiccation kinetics and heat shock experiments in open-top chambers	[135]
Microclimate: (temperature and relative humidity)	25 summer days	Multispecies	Data logger deployment	[225]
Air pollution	6-month transplanting field study	*Evernia prunastri*	Lichens as biomonitors: (1) lichen diversity; (2) bioaccumulation of trace elements, and (3) physiological status	[77]
Nitrogen addition	Field and laboratory experiments	*Usnea longissima, U. luridorufa, Ramalina calicaris var. japonica, U. dasopoga*	Determination of total N and P concentrations and pH	[72]

		and U. betulina		
hydrocarbon pollution	Literature survey	*Xanthoria parietina, Pseudevernia furfuracea, Evernia mesomorpha, Cladonia mitis, and Hypogymnia physodes*	Global bibliometric analysis	[226]
Heavy Metal Pollution	Field sampling	Multispecies	Metal detection through physiological Variables, e.g., chlorophyll damage, lipid oxidation, and protein content) analysis	[44]
Changing light conditions	Field sampling	*Hypogymnia physodes, Flavoparmelia caperata, and Parmelia sulcata*	Saturating light pulse and comprehensive analyses of fast and slow chlorophyll fluorescence transient (OJIP and PSMT) combined with quenching analysis	[69]

References

50. Chrást, P.; Zvěřina, O.; Komendová, R.; Barták, M. Focused study on metal allocation patterns in *Usnea antarctica* lichen from James Ross Island. *Marine Environmental Research* **2026**, *213*, 107614.

116. Boggess, L.M.; McCain, C.M.; Manzitto-Tripp, E.A.; Pearson, S.M.; Lendemer, J.C. Disturbance and diversity: Lichen species richness decreases with increasing anthropogenic disturbance. *Biological Conservation* **2024**, *293*, 110598.

223. Popovici, V.; Bucur, L.; Calcan, S.I.; Cucolea, E.I.; Costache, T.; Rambu, D.; Schröder, V.; Gîrd, C.E.; Gherghel, D.; Vochita, G.; et al. Elemental analysis and in vitro evaluation of antibacterial and antifungal activities of *Usnea barbata* (L.) Weber ex F.H. Wigg from Călimani Mountains, Romania. *Plants* **2022**, *11*, 32, doi:10.3390/plants11010032.

144. Worthy, F.R.; Schaefer, D.A.; Goldberg, S.D.; Wanasinghe, D.; Li, H.L.; Thiyagaraja, V.; Xu, J.C.; Wang, L.S.; Wang, X.Y. Simulated climate change impacts health, growth, photosynthesis, and reproduction of high-elevation epiphytic lichens. *Ecosphere* **2025**, *16*, e70224.

45. Jia, S.; Zhang, X.; Liu, Q.; Chen, Q.; Li, X.; Pang, X.; Li, J.; Wu, Q.; Zhao, L.; Liu, H. Spatial-temporal patterns of element concentrations in *Xanthoparmelia camtschadalis* transplanted along roads. *Pol. J. Environ. Stud.* **2020**, *29*, 121-129.

130. Alatalo, J.M.; Jägerbrand, A.K.; Chen, S.; Molau, U. Responses of lichen communities to 18 years of natural and experimental warming. *Ann Bot* **2017**, *120*, 159-170.

224. Gauslaa, Y. Changes in epiphytic lichen diversity along the urban-rural gradient before, during, and after the acid rain period. *Biodiversity and Conservation* **2024**, *33*, 2247-2263.

142. Borge, M.; Ellis, C.J. Interactions of moisture and light drive lichen growth and the response to climate change scenarios: experimental evidence for Lobaria pulmonaria. *Annals of Botany* **2024**, *134*, 43-58.

27. Taurozzi, D.; Gallitelli, L.; Cesarini, G.; Romano, S.; Orsini, M.; Scalici, M. Passive biomonitoring of airborne microplastics using lichens: A comparison between urban, natural and protected environments. *Environment International* **2024**, *187*, 108707.

195. Munzi, S.; Graça, C.; Martins, D.; Máguas, C. Differential response of two acidophytic lichens to increased reactive nitrogen availability. *Biologia* **2023**, *78*, 2049-2057.

140. Allen, J.L.; Lendemer, J.C. Quantifying the impacts of sea-level rise on coastal biodiversity: A case study on lichens in the mid-Atlantic Coast of eastern North America. *Biological Conservation* **2016**, *202*, 119-126.

135. Marín, C.; Barták, M.; Palfner, G.; Vergara-Barros, P.; Fernandoy, F.; Hájek, J.; Casanova-Katny, A. Antarctic lichens under long-term passive warming: Species-specific photochemical responses to desiccation and heat shock treatments. *Plants (Basel, Switzerland)* **2022**, *11*, 2463.

225. Haughian, S.R.; Burton, P.J. Microclimate differences above ground-layer vegetation in lichen-dominated pine forests of north-central British Columbia. *Agricultural and Forest Meteorology* **2018**, *249*, 100-106.

77. Paoli, L.; Munzi, S.; Guttová, A.; Senko, D.; Sardella, G.; Loppi, S. Lichens as suitable indicators of the biological effects of atmospheric pollutants around a municipal solid waste incinerator (S Italy). *Ecological Indicators* **2015**, *52*, 362-370.

72. Wang, M.; Wang, C.; Yang, L.; Guo, H. Impacts of short-term nitrogen addition on the thallus nitrogen and phosphorus balance of the dominant epiphytic lichens in the Shennongjia mountains, China. *Journal of Plant Ecology* **2019**, *12*, 751-758.
226. Iliquin-Inga, I.M.; Cortez-Lázaro, A.A.; Villanueva-Cadenas, D.I.; Rituay, P.; Arista, J.P.; Díaz-Valderrama, J.R. Bibliometric and systematic evaluation of lichens for biomonitoring in hydrocarbon pollution and mining. *Environmental and Sustainability Indicators* **2026**, *30*, 101172.
44. Yang, J.; Oh, S.O.; Hur, J.S. Lichen as bioindicators: Assessing their response to heavy metal pollution in their native ecosystem. *Mycobiology* **2023**, *51*, 343-353.
69. Osyczka, P.; Myśliwa-Kurdziel, B. The pattern of photosynthetic response and adaptation to changing light conditions in lichens is linked to their ecological range. *Photosynthesis research* **2023**, *157*, 21-35.
